# Evaluating the effectiveness of a mobile application to improve the quality, collection, and usability of forensic documentation of sexual violence

**DOI:** 10.1371/journal.pone.0278312

**Published:** 2022-12-14

**Authors:** Suzanne Kidenda, Roseline Muchai, Lindsey Green, Thomas McHale, Ranit Mishori, Brett D. Nelson

**Affiliations:** 1 Physicians for Human Rights, Nairobi, Kenya; 2 Physicians for Human Rights, Boston, MA, United States of America; 3 Georgetown University School of Medicine, Washington, DC, United States of America; 4 Divisions of Global Health and Neonatology, Department of Pediatrics, Massachusetts General Hospital, Boston, MA, United States of America; 5 Center for Social Justice and Health Equity, Department of Emergency Medicine, Massachusetts General Hospital, Boston, MA, United States of America; Zahedan University of Medical Sciences, ISLAMIC REPUBLIC OF IRAN

## Abstract

**Background:**

Survivors of sexual violence deserve timely and high-quality forensic examination, evidence collection, and documentation as part of comprehensive care. However, in many countries, the quality of medical-legal documentation is severely limited. MediCapt is an innovative digital application that enables clinicians to document forensic medical evidence as well as capture and securely store forensic photographs of injuries. This study evaluated the effectiveness and usability of MediCapt to document forensic medical evidence of sexual violence.

**Methods:**

This mixed-methods evaluation involved key-informant interviews, usability questionnaires, and forensic record reviews. Participants included clinicians, medical records personnel, information technology personnel, and health facility administrators, as well as law enforcement and legal professionals in Kenya.

**Results:**

The Physicians for Human Rights (PHR) data quality checklist found that using MediCapt led to significantly higher data-quality scores compared to paper-based forms. MediCapt forms scored higher on 23 of 26 checklist items. While a wide difference in quality was seen among paper-based forms, MediCapt appeared to both standardize and improve quality of documentation across sites. MediCapt strengths included data security and confidentiality, accuracy and efficiency, and supplemental documentation with photography. Weaknesses included infrastructure challenges, required technological proficiencies, and time to learn the new system. Although it is early to assess the impact of MediCapt on prosecutions, providers and law and justice sector professionals were optimistic about its usefulness. They identified MediCapt as appropriate for use with survivors of sexual violence and reported MediCapt’s legibility and photography features had already been commended by the court.

**Conclusion:**

MediCapt was well-received across all sectors, its use was perceived as feasible and sustainable, and it significantly improved the quality of collected forensic data. It is anticipated this improvement in forensic documentation will increase successful prosecutions, strengthen accountability for perpetrators, and improve justice for survivors.

## Introduction

Sexual violence is an urgent crisis that affects millions worldwide, impacting people of all genders, ages, and sexual orientation [1–3]. It is estimated that over 45% of women in Kenya will experience physical and/or sexual violence by an intimate partner or non-partner in their lifetimes [4–6]. Sexual violence is also a major contributor to a broad range of physical, psychological, social, legal, and economic consequences that adversely affect survivors, families, communities, and society at large [4,5,7].

The sexual and gender-based violence (SGBV) crisis in Kenya has been compounded by the onset of the COVID-19 pandemic. The Kenyan Ministry of Public Service and Gender issued a statement sharing concerns about the increase in SGBV in the country since the onset of the pandemic [8–10]. Its data showed that between January and December 2020, there was a 36% increase in cases reported to the national SGBV helpline compared to those reported the previous year. Similarly, a study by the National Crime Research Centre in Kenya showed a 92% increase in SGBV cases between January and June 2020 compared to the same period in 2019 [9].

Timely and high-quality forensic medical examination, evidence collection, and documentation are all part of comprehensive care for survivors of sexual violence and are crucial to ensuring survivors of SGBV can access justice [11]. High-quality documentation of the clinical exam after sexual assault has been shown to increase trial, prosecution, and conviction rates of perpetrators [12–15]. A South African study analyzed the association of sexual assault injury documentation and legal outcomes and found that conviction was more likely when cases had documented injuries [16]. In addition to legal justice outcomes, timely evidence collection may have other positive effects, such as empowering survivors, validating their experiences, and enhancing survivor agency [17,18].

The process of documenting forensic evidence of sexual violence is ideally standardized using forensic medical certificates, which are formal documents–sometimes issued by a government or court system—that trained clinicians use to systematically record forensic medical evidence for use in legal proceedings [19,20]. An evaluation conducted in Kenya found that a greater degree of medical evidence documented using the Post-Rape Care (PRC) form (Appendix 1)—the standardized medical certificate used in Kenya–was associated with an increased likelihood of an adjudication outcome favoring the survivor [13].

However, in Kenya and in many other resource-constrained contexts, there are reports of low-quality post-sexual assault medical-legal documentation as a result of numerous factors, including insufficient resources and gaps in training and support [21,22]. These, in addition to improper management of forensic evidence due to lack of consistent chain of custody mechanisms, remain some of the major factors in the significantly low number of convictions for SGBV, contributing to continued impunity [23]. Physicians for Human Rights (PHR), a global non-governmental organization, has been collaborating with partners in Kenya since 2011 to enhance local capacities for forensic documentation of sexual violence through training on forensic documentation, institutional assessment to strengthen post-rape care, and multisectoral network building to enhance collaboration amongst professionals. To help address persistent shortcomings in forensic documentation and to improve access to justice for survivors, PHR–in close partnership with colleagues in the medical, law enforcement, and justice sectors in Kenya, the Democratic Republic of Congo, and beyond–collaboratively designed and developed MediCapt, an award-winning application that enables clinicians to document medical evidence of sexual violence cases on a digital medical certificate (such as the PRC form in Kenya), capture forensic photographs of the injuries sustained, and store this crucial evidence securely in the cloud [24]. MediCapt was developed using a rights-based collaborative design approach that was guided by the Principles for Digital Development [25].

MediCapt was first introduced in Kenya in October 2017 with an initial roundtable with key stakeholders, a training in January 2018, and then the pilot testing period [26]. During the MediCapt pilot, the application development team received recommendations from the end users (health care professionals) on ways to make improvements to the application. Updates were made progressively during the pilot to enhance the usability of the application, based on end-user input, as well as to ensure that it was effective as a forensic documentation tool for this context.

This study evaluated the effectiveness and usability of the MediCapt mobile application as a tool to document forensic medical evidence of sexual violence. In particular, the authors sought to understand whether digital forensic documentation tools improve the evidence collection process and documentation quality compared to paper-based forensic documentation. As MediCapt is intended to be used in low-resource environments this study also endeavored to identify obstacles to uptake of digital forensic evidence tools as well as strategies to overcome them.

## Materials and methods

This mixed-methods study involved baseline and endline key-informant interviews, usability questionnaires, and forensic record reviews related to MediCapt implementation in Kenya. The evaluation was conducted at two hospitals in Kenya’s Nakuru County: Naivasha County Referral Hospital in Naivasha (trained in MediCapt October–December 2018) and Rift Valley Provincial General Hospital (RVPGH) in Nakuru (trained in MediCapt October 2020 –January 2021).

The study was conducted by a multidisciplinary team of professionals based in Kenya and the United States with experience in sexual violence research, forensic medicine, and assessment of health interventions in resource-limited settings.

### A. Data-quality assessments of forensic records

The Kenyan Ministry of Health’s PRC form is a two-page, triplicate form used by clinicians in Kenya to document survivor-reported sexual violence. The PRC form is divided into two sections: Part A, the description of the incident, the physical examination findings, and the documentation of the clinical management and forensic evidence; and Part B, the psychological assessment.

The MediCapt application (S1 and S2 Figs) collects the same data as the paper-based PRC forms. The application also allows providers to obtain (with survivor consent) forensic photographic evidence alongside the traditional history-and-physical data.

To objectively assess the data quality of the paper-based and MediCapt forensic records, a data quality checklist tool was iteratively developed by the study team with local clinicians, law enforcement, and legal professionals in Kenya. The draft checklist was piloted and underwent an inter-rater reliability study in which two independent researchers scored 31 de-identified MediCapt records [27]. For nearly all 26 checklist items, there was very strong inter-rater agreement; the one item with “moderate” agreement was the checklist item related to chief complaints, suggesting greater subjectivity in assessing these [27].

Kenyan data collectors were recruited and trained on use of the finalized checklist tool. Paper-based records were selected and reviewed at each of the two facilities. At each facility, five paper records per month (or all records in the rare months that had less than five paper records) were randomly selected for the 20 months preceding MediCapt training, for an estimated total of 100 paper-based records per site. This purposive stratified sampling by month helped ensure the sample was representative and minimize sample and selection biases.

For quality assessment of MediCapt records, a spreadsheet of de-identified MediCapt output for all completed MediCapt forms from both facilities was obtained from PHR.

Data quality scores for paper-based and MediCapt records were then compared. Given its ability for offline data collection and the PHR team’s familiarity with the software, Kobo Toolbox (Cambridge, MA) was selected for data collection [28]. Data were analyzed using traditional descriptive analysis (e.g., means, standard deviations) and non-paired two-tailed t-tests comparing data quality checklist scores for paper-based versus MediCapt forensic records. Statistical significance was set at p<0.05.

### B. MediCapt usability questionnaires

To evaluate the usability and feasibility of the MediCapt application in these settings, a 10-page usability questionnaire (Appendix 2) was administered to local health professionals who had been trained on and had used MediCapt for forensic documentation [27].

The questionnaire consisted of both open- and closed-response questions. Open-response answers were organized into general themes and closed-response questions were analyzed using traditional descriptive analyses. The software Dedoose Version 4.12 (Los Angeles, CA) was used for coding and code analysis [29].

### C. Key-informant interviews

Participants included clinicians, law enforcement, judiciary professionals, information technology professionals, and medical records personnel who interact with PRC and MediCapt forms and had been trained (from October 2018 to January 2021) or oriented on the MediCapt application.

Semi-structured interviews were conducted at each of the two sites at the evaluation’s baseline (October 19–23, 2020) and endline (June 21-July 8, 2021) with clinicians, medical records, administration staff, law enforcement professionals, and legal professionals. Law enforcement and legal professionals, such as police officers, prosecutors, and magistrates, who participated in previous MediCapt sensitization sessions were also interviewed.

A semi-structured interview guide (Appendix 3) was developed and iteratively revised by the PHR team for use during the interviews. This guide was used for both the baseline and endline assessments. Consent was obtained from participants to take part in the study and for evaluators to digitally record all interviews. Verbatim transcriptions of these recordings were made and de-identified of any participant or survivor names or other identifying information. These transcripts subsequently underwent theme analysis with Dedoose [29] by two independent researchers using an inductive approach to identify emergent themes.

Ethical review and approval were obtained from the institutional review boards of Georgetown University (Washington, D.C., U.S.; IRB ID# STUDY00001945) and Egerton University (Njoro, Kenya; Protocol #EUREC/APP/099/2020).

## Results

### A. Data quality assessment of forensic records

The quality of 197 paper-based PRC forms and 139 MediCapt forms was evaluated using the validated data quality checklist. When compared, MediCapt forms more frequently had higher data-quality scores than paper-based forms (Table 1). MediCapt was associated with higher scores in 88.5% (23 of 26) of checklist items. The mean score for paper-based forms (n = 197) was 42.1 (SD 6.2, range 5–52), with 81 forms (41.1%) achieving the target score of 44 out of 54 (>80%). The mean score for MediCapt forms (n = 139) was 48.2 (SD 6.8, range 36–53), with 133 forms (95.7%) achieving the target score of 44 out of 54 (>80%). This difference in quality between the two form types was statistically significant (t-value -11.0, p-value <0.00001). Overall, there was a total of 336 forms, with a mean data-quality score of 44.7 (SD 5.8).

**Table 1:** Quality of documentation in paper-based versus MediCapt forms. Cells highlighted in gray represent the higher quality score between the two types of forms. Each item is scored from 0–2, except for Item 24, which is scored from 0–4.

	Average score		
	Paper-based forms (N = 197)	MediCapt forms (N = 139)	All forms (N = 336)
**DEMOGRAPHICS**			
1. All 4 dates (dates of form, birth, exam, incident)	1.91	1.95	1.93
2. Three names of survivor	1.76	1.86	1.80
3. Survivor contact info (address and phone)	1.44	1.58	1.50
4. Orphans and vulnerable children (OVC) status	1.89	1.57	1.76
**HISTORY**			
5. Perpetrator info (gender, est. age or adult/non-adult, unknown/known)	1.76	1.92	1.82
6. Chief complaints	1.50	1.64	1.56
7. Circumstances surrounding incident	1.44	1.50	1.46
8. Previous reporting and care	1.78	1.93	1.84
**PHYSICAL EXAMINATION**			
9. Notations on body map	1.92	1.96	1.93
10. Statement in “Comments” summarizing body map exam	1.71	1.86	1.77
11. Date of last consensual intercourse	1.54	1.48	1.52
**FORENSIC**			
12. Clothing info (4 fields)	1.65	1.99	1.79
13. Toilet and bathing info (2 fields)	1.86	1.99	1.91
14. Info on perpetrator marks	1.74	1.81	1.77
**GENITAL EXAMINATION**			
15. Genital exam info	1.88	1.95	1.91
16. Statement in “Comments” summarizing genital exam	1.38	1.68	1.50
**MANAGEMENT**			
17. Management info	1.94	2.00	1.96
18. Referral info	1.82	1.94	1.87
**LABORATORY SAMPLES**			
19. Labs sent	1.95	2.00	1.97
**CHAIN OF CUSTODY**			
20. List of chain-of-custody samples	0.08	1.83	0.80
21. Examining officer signature and date	1.86	1.97	1.90
22. Police officer signature and date	0.16	0.12	0.14
23. Document signed by examining officer within 48 hours of patient visit	1.76	1.93	1.83
**PSYCHOLOGICAL ASSESSMENT (PART B)**			
24. Part B (including child section if relevant)	1.81	3.84	2.65
**GENERAL**			
25. Writing legible?	1.79	1.99	1.87
26. Content understandable? (e.g., clear meaning, avoids unexplained medical jargon, etc.)	1.81	1.97	1.88
**TOTAL SCORE (out of 54)**	Mean* **42.1 (78.0%),** **SD 6.2**	Mean* **48.2** **(89.3%),** **SD 6.8**	**Mean** **44.7** **(82.7%),** **SD 5.8**
	(t-value -11.0, p-value <0.00001)		

Comparing the two sites, the quality of the paper-based data was statistically different. The paper-based data in Naivasha (n = 97) had an average data quality score of 40.1 (SD 4.0), while the paper-based data in Nakuru (n = 100) had a statistically higher average data quality score of 44.1 (SD 7.2) (t-value -4.82, p-value <0.00001).

The quality of the MediCapt data was statistically the same across sites. The MediCapt data in Naivasha (n = 91) had an average data quality score of 48.3 (SD 2.7), while the MediCapt data in Nakuru (n = 48) had an average data quality score of 48.1 (SD 2.4) (t-value 0.39, p-value 0.70).

Table 1 shows the quality of documentation in paper-based versus MediCapt forms. The checklist items for which MediCapt forms scored particularly high relative to the paper-based forms were clothing information (#12), statement in “Comments” summarizing the genital examination (#16), list of chain-of-custody samples (#20), and psychological assessment (Part B) (#24).

The three items for which the paper-based forms scored higher than MediCapt were the orphan-vulnerable children (OVC) status of the survivor (#4), date of last consensual intercourse (#11), and police officer signature and date (#22). The police officer signature and date prompt was also one of the two checklist items that tended to have the lowest data-quality scores across both types of forms, the other being the circumstances surrounding the incident (#7).

### B. MediCapt usability questionnaires

Fourteen individuals completed the MediCapt usability questionnaire. These included clinical officers (n = 8), nurses (n = 5), and a gender officer / social worker (n = 1). Table 2 shows the results of the closed-response portion of the MediCapt usability questionnaire.

**Table 2:** Results of closed-response portion of the MediCapt usability questionnaire.

Position	Clinical Officer (8), Nurse (5), Gender Officer/SW (1)
	**Average**
Years conducting sexual assault examinations	5.4 (range 2–11)
Number of sexual assault cases entered into MediCapt	11.2 (range 1–30)
Approximate number of sexual assault examinations typically conducted each month	11.4 (range 1–45)
Approximate number of minutes spent per sexual assault examining the survivor (not including documentation)	25.7 (range 10–60)
Approximate number of minutes spent per sexual assault documenting using the PAPER FORM	32.7 (range 10–120)
Approximate number of minutes spent per sexual assault documenting using MEDICAPT	36.8 (range 5–60)
	**Yes**
Ever used a mobile phone	100%
Ever used a smart phone	100%
If you have experience using a smart phone, what have you used it for?	
Communicate with family and friends	100%
Look up information online	100%
Check email	100%
Take pictures	100%
Assist with my clinical work	64%
Find information to make medical decisions	86%
Take notes	71%
Play games	57%
Use apps	79%
Listen to music	79%
Experience using applications, or “apps,” on smart phones	100%
Ever used the camera function on a mobile phone or smart phone	100%
Ever used a digital camera (not on a mobile or smart phone) to take a photograph	100%
Normally take forensic photographs when you conduct sexual assault examinations (before using MediCapt)	0%
**Agreement scores for various statements (listed from strongest disagreement to strongest agreement)**	
The use of MediCapt with a sexual violence patient is culturally unacceptable.	-1.3
I have to rely on a generator to charge smart phones or tablets on a daily basis.	-1.1
The tablet should be smaller.	-0.9
I currently complete a paper-based medical certificate for examinations of all sexual violence patients.	-0.9
I find the text size on the screens too small.	-0.9
The process of obtaining consent to use MediCapt for data collection is too cumbersome.	-0.9
I am likely to lose my device.	-0.7
I think connection to the internet is a major problem for uploading files.	-0.7
It is difficult to charge the smart phones or tablets on a daily basis.	-0.7
The printer is likely to be stolen.	-0.7
It is difficult to maintain printer supplies (ink, paper, etc.).	-0.6
It is difficult to get reliable Wi-Fi or internet access to transmit the files.	-0.4
The device is likely to get stolen.	-0.3
The printing process with MediCapt works well.	0.1
I have had enough training to use MediCapt correctly.	0.2
MediCapt helps me save time in conducting sexual assault examinations.	0.4
The printer is likely to be used by others for purposes UNRELATED to MediCapt.	0.4
It is easy to use MediCapt while I am conducting a sexual assault examination on a patient.	0.5
MediCapt helps me save time in documentation.	0.6
It is easy to troubleshoot problems that I encounter with MediCapt.	0.7
It is easy to connect the Bluetooth keyboard to the tablet.	0.8
It is easy to type using the keyboard.	0.8
I think that sexual violence patients accept my use of MediCapt during their examination.	0.8
It is easy for me to hold the tablet.	0.9
It is easier for me to take forensic photographs using MediCapt.	0.9
Printing the MediCapt document serves the patient well.	0.9
My colleagues will be happy using MediCapt.	0.9
Patients readily provide consent for MediCapt to be used in documenting their cases.	0.9
Printing the MediCapt document is more acceptable to me than sending the data electronically.	0.9
I think that the forensic photography function on MediCapt makes it more comfortable for my patients to be photographed, versus using a separate camera to take photographs.	1.0
I believe that my patients understand the risks and benefits of using MediCapt.	1.0
Additional measures will need to be put into place to make sure this device gets used.	1.0
Health care professionals who use MediCapt will take better forensic photographs because they are using MediCapt.	1.0
I have Wi-Fi or other internet access in my community or health care center.	1.0
I have access to reliable electricity in my community or health care center.	1.0
When I encounter a problem with MediCapt, I know who to turn to for help.	1.1
When I encounter a problem with MediCapt, I am satisfied with the help I receive.	1.1
I like the colors used on the screens for MediCapt.	1.1
I find that multiple places to enter information on a single screen makes data entry on MediCapt easy to use.	1.1
The different screens all made sense to me.	1.1
I like the prompts to take forensic photographs that were built into MediCapt.	1.1
I feel confident in my ability to explain to the patient the purpose and risks involving the use of MediCapt to obtain and record their information.	1.1
MediCapt is intuitive to my needs when documenting sexual assault examinations.	1.1
I am able to take forensic photographs easily using MediCapt.	1.1
It is easy to type on the tablet.	1.2
It is easy to take photographs using the tablet.	1.2
I find it easy to transition from one screen to the next.	1.2
I find the pictogram easy to use.	1.2
It was easy for me to use MediCapt.	1.2
Using MediCapt makes a difference in survivor’s cases.	1.2
The training on using MediCapt with the patient helped me incorporate it into practice.	1.2
Overall, I am satisfied with MediCapt.	1.2
My patients will be better served if I use MediCapt.	1.3
I am comfortable using MediCapt in my clinical practice.	1.3
I like to use new types of technology to help my patients.	1.3
MediCapt will ensure that sexual assault records are transferred to the appropriate law enforcement and legal personnel.	1.3
MediCapt is better than what I am currently using to document sexual assaults.	1.3
The MediCapt app “made sense.”	1.3
It is easy to use the touchscreen on the tablet.	1.4
The risks to the patient of lost personal information are greater with the paper form than with MediCapt.	1.4
I could one day train my colleagues on how to use MediCapt to document sexual assault examinations.	1.4
MediCapt offers a useful way to take forensic photography.	1.5
The screens appear to be straightforward and easy to use.	1.5
The tablet itself appears to be suitable to document sexual assault examinations.	1.6
MediCapt helps me do a better job of documenting sexual assault examinations.	1.6
I am obtaining the consent of all patients prior to using MediCapt.	1.7

On average, respondents had 5.4 years (range 2–11) of experience conducting sexual assault examinations. Theyconduct 11.4 examinations (range 1–45) each month. They had each used MediCapt for an average of 11.2 times (range 1–30).

Respondents reported that, at least initially, using the MediCapt form may take slightly longer than using the paper form. Questionnaire participants reported spending an average of 32.7 minutes (range 10–120) documenting with the paper PRC form and 36.8 minutes (range 5–60) documenting with MediCapt. Subsequent interview data presented in the next section suggest that this trend is reversed as providers became more familiar with the MediCapt application. For the examination of the survivor, participants reported spending an average of 25.7 minutes (range 10–60) using either of the forms.

All participants were familiar with smart phones and typically use them for communication, looking up information, checking email, and taking pictures. No respondents had taken forensic photographs prior to using MediCapt.

Usability questionnaire respondents generally agreed that the tablets are useful and MediCapt is easy to use. Participants agreed that MediCapt is appropriate for use with survivors of sexual violence, MediCapt is acceptable to providers and survivors, and using MediCapt in these settings is both feasible and sustainable.

### C. Key-informant interviews

Semi-structured key-informant interviews (n = 57) were conducted during this evaluation of the MediCapt program. The mean years of work experience among interviewees was approximately 10.5 years (range 0.25–30). Additional demographic information about the interview participants is presented in Table 3.

**Table 3:** Semi-structured interview participants conducted during the baseline (N = 24) and endline (N = 33) assessments.

	Baseline assessment n (%)	Endline assessment n (%)
**Location**		
Naivasha Sub County Hospital	12 (50.0)	17 (51.5)
Rift Valley Provincial General Hospital GBVRC	12 (50.0)	16 (48.5)
**Profession**		
Clinical/medical officer	9 (37.5)	11 (33.3)
Nurse, nurse administrator	5 (20.8)	7 (21.2)
Nursing officer	4 (16.7)	3 (9.1)
Health records officer	3 (12.5)	0 (0)
Information and communications technologist	1 (4.2)	4 (12.1)
Gender officer	1 (4.2)	1 (3.0)
Mental health professional	1 (4.2)	1 (3.0)
Legal professional	0 (0)	4 (12.1)
Law enforcement professional	0 (0)	2 (6.1)
**MediCapt trained?**		
Oriented (e.g., some law enforcement and legal professionals)	15 (62.5)	6 (18.2)
Formally trained	9 (37.5)	27 (81.8)
**Years of experience at facility**		
Mean	10. 6	10.5
Range	0.25–30	1–30

Of the interviewees who participated in the baseline assessment (n = 24), 19 (79%) were available to participate in the endline assessment (n = 33). Of the 19, there were 15 (79%) clinicians and four (21%) non-clinicians (i.e., medical records/administration staff). The baseline did not have participants from the judiciary, prosecution, and law enforcement officers, the endline assessment engaged six individuals from these sectors.

By design, the baseline assessment interview guide largely focused on the paper-based PRC forms. The endline assessment interview guide largely focused on the MediCapt application and how its use compared to that of the paper-based PRC forms. The codes that emerged from the interview transcripts were organized into five themes summarized in Table 4.

**Table 4:** Themes from key-informant interviews, listed by frequency at baseline, endline, and total, along with illustrative quotations as relevant.

Theme 1: Strengths of paper forms	Frequency totals		
	Baseline	Endline	Total
Paper form works well	29	0	29
	*“It’s*.* *.* *.*very effective*, *well explained*, *and direct and it really helps in making it easier for when*.* *.* *. *you are recording the findings*.*”–Clinician*:		
Paper form is in triplicate	18	1	19
	*“The good thing with those forms is that they are triplicate*.*”–Clinician*		
People already familiar with paper form	10	2	12
	*“We have been using it [the paper form] for quite a while*, *and we are so much used to it*.*”–Clinician* *“Most of the advocates maybe within the [Nakuru] region are aware of the new … [MediCapt application]*. *But I think it’s not in some other regions*, *because sometimes you may find an advocate from maybe Kericho*, *a different region*, *who has never seen the [application]…*. *But as time goes by*, *they will be made aware of the same*. *Of course*,.* *.* *. *when you say ‘PRC forms*, *the old ones*,*’ everyone knows the document*.*”–Prosecutor*		
Captures most of the details	8	1	9
Paper form not easily lost	8	0	8
Paper form is concise and easier to present in court	5	2	7
	*“An advantage with paper-based PRC form[s] is that when I’m presenting these PRC forms in court*, *it’s very easy for me to present the case*. *Like for instance*, *I will give a scenario where there was a time I … presented the case in court severally with the PRC and with the MediCapt*. *In that*, *the challenge we generally have with MediCapt … is that we generally have a lot of paperwork*, *a lot of them*. *In that*, *when you go to the magistrate*, *he tells you*, *kindly can you present what is important*, *and you have like 10 pages*. *So now you start perusing*, *looking for what is important to you*, *and you don’t know whether it is important to the judge*.*”–Clinician* *“You see the advantage with the other one [paper PRC]*, *the hard copy*, *it is easier [to present in court]*. *You just get one sheet*, *and you know each section*. *It’s one sheet*, *not many sheets*, *just one*. *And mostly these magistrates and prosecutors*, *they just tell you*, *‘Read what is important*.*’”–Clinician*		
Paper form’s diagrams are helpful	5	0	5
Paper form navigation is easy	3	2	5
	*“The main problem with MediCapt compared to PRC is it has many pages to go through*. *The old PRC we find it is easy to navigate for us as police*, *the other one has too many pages so going through is a challenge*. *But for PRC*, *it is very much in order*, *it carries a lot of information*. *You see it carries information from the doctor which we are able to understand*, *and it make us able to open a file and to take the perpetrator to court*. *It gives us direction*.*”–Police officer*		
Paper form has plenty of room	4	0	4
Paper form has guidelines	3	0	3
	*“Another advantage is*, *on the paperwork*, *we normally have some subheadings*, *the circumstance surrounding the survivors whatever*, *we have those subheadings*.*”–Clinician*		
Paper form is well organized	3	0	3
Paper form can be completed collaboratively	3	0	3
	*“Sometimes when it is paperwork*, *you might find maybe someone was [working] at night*, *they came and filled in the PRC*, *… they leave having left some gaps*, *maybe the labwork was not ready by the time [they’re] leaving*. *And I come in*.*…You know for paperwork*, *I just continue then [completing the paper form] and at the down part I will sign*.*”–Clinician*		
Paper form can be completed quickly	2	0	2
Paper form is easily accessible	2	0	2
Paper form is easily updated	2	0	2
Paper form is confidential	1	0	1
Paper form is customized w/logo etc.	1	0	1
**Theme 2: Weaknesses of paper forms**	**Frequency totals**		
	**Baseline**	**Endline**	**Total **
Paper form can be lost	57	17	74
	*“We told them the PRC form may actually disappear*, *might be stolen*, *but when we have the application*, *their data is stored there permanently*. *Nobody can take it away*.*”–Clinician* *“Before MediCapt was rolled out*, *we were using the archaic PRC forms*. *That form was very big*, *it had to be folded into two*, *and it tended to lose data because of the folding and at times…*. *You see it is not us who in the first place who report it*, *it is the police*. *And then by the time it reaches to us*, *it is many days down the line so… one could not read it*.*”–Magistrate*		
Others can change or destroy paper form without permission	33	4	37
	*“The police at times might collude with the accused person to create some doubts*. *The police are very smart*: *you just need to pour water on the original PRC form*, *and it cannot be read*, *so in [the documentation of] penetration*, *you cannot prove that there was penetration*. *But now with the system that you have rolled out [MediCapt]*, *if they went ahead and did that and introduced such a thing before me*, *I would just adjourn the case and tell them to come back tomorrow*, *go for another copy [of the MediCapt report]*. *So according to me*, *[with] this roll out [of MediCapt]*, *the victim will get justice because the [MediCapt] PRC form is clean in the first instance*, *unlike in the past documents*.*”–Magistrate*		
Paper form is not confidential	22	7	29
	*“For PRC form one thing*, *the privacy and confidentiality of information is not well observed*, *because the PRC form will be just filed and like in our office*, *we don’t even have a lockable place so we can’t lock them*.*”–Medical records officer* *“I find papers lying around and I’m like*, *no*, *this is not right*.*”–Clinician* *“First [concern with the paper-based PRC forms]*, *confidentiality*. *You know*, *with the previous papers*, *anybody could access [them]*. *But with MediCapt*, *it is really safe as confidentiality and privacy is really enhanced in MediCapt*.*”–Clinician*		
Paper form has limited space	13	3	16
Paper form is a lot of work / takes a lot of time	12	1	13
Difficult to correct or modify paper form	12	1	13
Paper form has missing or incorrect data	11	2	13
	*“Though the challenge is usually that sometimes some of the clinicians would fill and they leave blanks you know it’s not like the MediCapt where you cannot submit before you fill everything*, *so if they decide not to complete*, *that is usually the major challenge when it comes to documentation [on] the paper-based [forms]*.*”–Clinician* *“Major difference is that there are no blank spaces being left in MediCapt compared to paper work*, *and then especially when it comes to time and date of incidence*, *you know people used to leave that place blank*, *which was also becoming a challenge*.*”–Clinician* *“And then sometimes you find dates discrepancy*, *a PRC form was filled before even the incident occurred*, *those are some of the challenges we were getting and now they have reduced*.*”–Clinician*		
Paper form doesn’t have consent form	4	7	11
	*“[With the] PRC form*, *[consent form] was not attached*. *In fact*, *rarely*, *let me confess*, *we never used to fill the consent forms*. *It was only the patient comes*, *you go direct[ly to the PRC form]*, *you just get a verbal consent*. *But you see with the [MediCapt] app*, *they have to sign [the consent]*. *That is the beauty of everything because now*, *as far as even legal issues are concerned*, *nobody can go against what they have signed*.*”–Clinician*		
Printed paper forms are not available	9	0	9
Writing in paper forms can be illegible	0	8	8
Paper form’s carbon copy is unclear	4	3	7
Paper form text is too small	5	0	5
Part B of the form is difficult to complete	4	1	5
Paper form is disorganized or challenging	4	0	4
Paper form book is bulky	2	1	3
** Theme 3: Strengths of MediCapt**	**Frequency totals**		
	**Baseline**	**Endline**	**Total **
MediCapt securely stored / data not lost	22	77	99
	*“Anytime the clients would lose the documents*, *you can always retrieve the documents again so you are sure that the documents are there*, *the documents are safe*, *they are not getting lost because even if they lose the copy that they have*, *even if the copies with the police is lost*, *you can always come and print again*, *because the documents are well stored*. *So digital is better*, *digital is better*.*”–Clinician* *“I think it will impact positively*, *noting that the victim can have the form lost or sometimes someone can even pluck it from a file*, *they can pluck the P3 form (The Kenya Police Medical Report Form*, *used by the police to document forensic medical evidence for all crimes)*. *You know not everywhere people are good*. *So when it is plucked and I have the original file*, *I can be able to now to send the victim back to the hospital and get another copy because it is there in the system*. *So we now have preservation of evidence*. *So that those ones who want to kill the wheels of justice they will not be able to*.”–*Prosecutor*		
MediCapt is more time efficient and convenient	10	36	46
	*“The benefits we are able to see is that we have efficiency*. *We were hoping that one time we would be able to get tangible evidence*, *we will be able to print and see what we are presenting and well*, *you are now able to describe what you have photographed*. *It’s like work has become easy to perform*.*”–Nursing administrator*		
MediCapt stored data easy to access / available to other sectors	5	35	40
	*“Other authorized organizations like the judiciary*, *the security [i*.*e*., *the police]*, *they can access some data*. *But only what they need from that particular information*, *the only information that is required by them*. *So*, *there is*, *no one will be able to get what is not required and use it for other purposes*.*”–Clinician* *“It can be accessed by other users even other places to get the information but there is a privacy on how you can access the same material*.*”–Clinician*		
MediCapt can take photos	4	31	35
	*“I think MediCapt is good*, *way much better considering right now they are even taking the photos of the injuries while it has already happened*, *immediately*. *There is preservation of evidence way better than the PRC*.*”–Prosecutor* *“Photographic evidence is good because sometimes when the victim says she had been burnt in the face and it was taken then later on they heal*, *and the victim wants to forgive the perpetrator*, *you see you can take them back and show how they were previously*, *and the danger they were in*. *So it is easier even to convince her and to tell her not to withdraw the case*.* *.* *. *because we have such cases*. *And also*, *for the prosecutor*, *when the prosecutor sees previously how the victim was and how right now she is healed and she says she wants to withdraw the case*, *it can guide the prosecutor not to agree to withdraw that case*. *So that the victim may get justice also*. *It’s good for prosecution and also the court as it can also inform the court how the injuries were*. *Because sometimes the prosecutor may say they want to withdraw but when the court also sees how the victim was*, *the court can also reject that application noting the degree of injuries that were there on the victim*.*”–Prosecutor*		
MediCapt is more confidential	8	26	34
	*“For me digital is the best*, *because like you see when you have the tablet you find that only us who are trained know the password*, *and you see all those who are trained are health care workers who understand the confidentiality and privacy of that information*, *so it’s not easy to be accessed by anyone*.*”–Medical records officer*		
MediCapt won’t allow skipped sections	14	16	30
MediCapt requires consent completion / good consent process	0	29	29
MediCapt is more comprehensive / accurate	2	24	26
	*“You see the accuracy of it now is not comparable and it’s acceptable*. *More acceptable than I think it was before*.*”–Clinician*		
Difficult to tamper with MediCapt form	11	9	20
MediCapt improves services for survivors	0	16	16
All medical records should or will be digital	7	7	14
MediCapt stores more data / more room for answers	6	8	14
MediCapt reports are more legible	0	11	11
Copies of completed form can be printed	0	10	10
MediCapt is more readily available than paper	4	4	8
MediCapt data can be stored easily	5	2	7
Printer works well	0	3	3
MediCapt allows errors to be corrected	2	0	2
**Theme 4: Weaknesses of MediCapt**	**Frequency totals**		
	**Baseline**	**Endline**	**Total **
Internet / logging in / connection issues	0	36	36
	*“The Wi-Fi here sometime is a challenge and we only use the Wi-Fi from the hospital*. *So when the Wi-Fi is off*, *we really cannot do anything*.*”- Clinician*		
MediCapt report length and format is challenging	6	29	35
	*“I’m just mentioning this from the court’s perspective*. *You just find that the magistrate has like 40 cases*, *so he’ll just tell you because he wants to make a ruling to just use the least time possible*, *and whenever somebody attacks you from that direction*, *you just feel frustrated*. *But with the hard copy*, *if I look at the name*, *I’m seeing even the results from the other side*.*… [T]he hardcopy printouts from MediCapt …*, *they are bulky*. *The challenge is*, *if I try to read [it in court]*, *you find you peruse a lot of pages*, *you waste a lot of time*, *confidence takes a hit*, *and the magistrate is saying harakisha [speed up]*, *and the perpetrator there is smiling*, *and you find you are fighting a losing battle*. *Because these perpetrators normally get some lawyers in the remands*, *they are really armed with questions*., *well-armed*.*”–Clinician*		
There are issues with the printer	3	31	34
	*“I can say at least they can bring a big printer*, *you know this one is small*, *so when you send*, *it is too slow*, *it takes time*. *Not like the other printer*, *the older one was smaller and was very slow*, *this one the speed has improved but still slow*. *And there are a number of pages to print*, *they are normally nine of them [pages]*.*”–Medical records officer*		
MediCapt requires more time and effort to complete	4	26	30
Problem with MediCapt form itself	0	27	27
Difficulty getting consent for photos	0	23	23
	*“Obtaining consent*, *and especially on the photo part*. *They are very…*. *They don’t want their photos to be taken … because they see it’s an online thing*, *they think their information is going to go online*. *Most of them are not comfortable with that*, *especially the under-18[s] because they are brought by their parents*, *their parents don’t want that*. *For me*, *that is the major problem*.*”–Clinician* *“They are reluctant*. *I have encountered situations where they refuse to provide consent for photography*, *especially for the minors*.*”–Clinician*		
MediCapt requires typing proficiency	3	15	18
MediCapt requires providers and patients learning new approach	2	8	10
	*“One thing about the MediCapt is*, *since the public does not know about it*, *they feel the doctor is doing something different*. *Sometimes you are force[d] to use it while they see so that they can be able to know that you are not watching a movie*, *you are not on Facebook*, *social media*, *and such*. *Some of them are a little bit reluctant with the photography part*. *Because they say that it is my photograph–suppose I see it elsewhere*, *I will be embarrassed*.*”–Clinician*		
Difficulty connecting Bluetooth keyboard to tablet; connection/IT issues	0	9	9
	*“Our keyboards have a problem*, *actually we use a typing board for the tablet*, *not the key board*, *we [are] directly typing on the tablet*. *The challenge is connecting the Bluetooth to the tablet*, *that part is a problem*. *Sometimes the IT don’t know how to solve it sometimes*.*”–Clinician*		
MediCapt is not available overnight	0	6	6
MediCapt passwords are forgotten	0	6	6
Photography violates survivor’s privacy	0	6	6
Need greater security from hacking	0	5	5
MediCapt has duplicate questions	0	4	4
Difficult to write signature on tablet	0	3	3
Device could potentially be damaged	0	3	3
Difficulty taking photos	0	3	3
MediCapt use was stopped for a while	0	3	3
Photography should be removed since not needed by judge	0	3	3
Difficult to complete MediCapt collaboratively	2	0	2
Battery can run out on device	0	2	2
Will police and court accept digital records?	1	0	1
** Theme 5: Other issues**	**Frequency totals**		
	**Baseline**	**Endline**	**Total **
More training and awareness needed	27	42	69
	*“There are some new staff who have joined us*, *and they are quite good and they are trained*. *So*, *if we can get time when any other groups are being trained*, *they can also come in*. *When we are a big number*, *you can be certain that one of us must be on duty and will be able to capture the details*.*”–Clinician* *“The only issue that we are having especially here is that with the MediCapt*, *it has brought the notion that so-and-so is the one who is supposed to handle the sexual defilement gender violence cases since they are the ones with the tablet*. *So*, *if you find that when survivors come*, *they have to wait for a certain person*. *Yet everybody should be able to handle the case*.*”–Clinician* *“I would suggest more training on the same … so that people can know much about it because I understand not many know about it*. *The magistrate did not know about it*, *I knew about it*, *but my colleagues also did not know about it*. *But I’ve informed them*, *but other [police] stations*, *I don’t know whether they have the benefit of knowing about that*.*”–Police officer*		
We are able to obtain consent	0	33	33
	*“We don’t have any issues with the consent*, *as far as we’ve explained the procedure to the patient and we explain as to why we are using the tablet*, *they are okay with it*.*”–Clinician*		
Gratitude for training and support	4	10	14
	*“We are really happy about the support you [PHR] have given us*. *As a hospital*, *I don’t know whether we would get to that stage we are in today without you*. *And this one has gone quite a mile in helping the community who actually cry for justice every now and then when such events occur*.*”–Clinician*		
More staff needed / heavy workload	5	8	13
Never used or not currently using MediCapt application	0	11	11
Providers need to use MediCapt more	0	9	9
Long delays before court hearing	9	0	9
Multi-sector collaboration	4	2	6
More tablets needed	0	5	5
PHR should focus more on boy survivors	3	2	5
Request for provider guide at start of MediCapt	0	4	4
Redundancy between PRC and P3 forms	3	0	3
Digital records is forward looking	0	2	2
Request to have MediCapt on a computer	0	2	2
Clinician completing form not available in court	2	0	2
Limited SV services	2	0	2
Infrastructural improvements needed	1	0	1
SGBV research is needed	1	0	1

There were multiple features of the paper-based forms that interview participants appreciated (Table 4: Theme 1). The leading reported strengths of the paper form were its triplicate design and that it simply “works well.” Participants also indicated that there was existing familiarity with the forms among people across all sectors. Respondents stated that paper forms capture most of the necessary details, which made them easy to review and navigate when pursuing cases.

Interview participants cited several weaknesses of paper forms (Table 4: Theme 2). The most frequently reported weaknesses were that the paper forms can be lost, changed or destroyed without permission and are not confidential. Logistically, participants also noted that the paper-based forms have multiple features that complicate their use, including limited space, requiring a lot of time to complete, being challenging to correct, often having missing or incorrect data, and not having a survivor consent section.

Many interview respondents felt the digital documentation tool had significant strengths and could address many of the weaknesses of paper-based documentation. The most frequently reported strengths of MediCapt (Table 4: Theme 3) included elements related to data security and completeness of the data collected. Respondents appreciated MediCapt’s secure storage, ease of accessing stored data across sectors, confidentiality, and that tampering with the documentation was difficult. MediCapt assisted in the completeness of data collection with its greater time efficiency and convenience, ability to take photos, inability to skip questions (hence, enhancing completeness), required consent process, and overall comprehensiveness and accuracy.

Respondents were asked what areas of weaknesses they had noted with the MediCapt application (Table 4: Theme 4). The most frequently reported MediCapt weaknesses related to information technology issues included internet or logging-in difficulties, printer troubles (such as being inaccessible, slow, or jamming), and the requirement for typing proficiency to use MediCapt. Respondents also noted challenges with the length and format of the form printed from MediCapt, leading to difficulty presenting the form in court. Participants also shared that completion of MediCapt required more time and effort, and specific issues with the MediCapt form itself, such as small font, unclear format, and some repetition. Some respondents also noted difficulty obtaining consent for photography with MediCapt.

In addition to strengths and weaknesses of the paper forms and MediCapt, interview participants discussed several other relevant issues (Table 4: Theme 5). Most frequently, this included a request for more PHR/MediCapt training. Also, in contrast with some of the above interview data, but consistent with the usability questionnaire findings, most providers reported they were able to obtain consent from survivors using the MediCapt application–even consent for photography.

## Discussion

This study demonstrates that MediCapt–a mobile application for the forensic documentation of sexual violence–more accurately, consistently, comprehensively, and securely collects the same forensic medical certificate data collected by the paper-based PRC forms in Kenya. In addition, the application has the unique ability to collect forensic photographic evidence that is not possible with the paper-based form. This study also shows that this mobile application is acceptable to the end users in the health, law enforcement, and legal sectors in Kenya. There is optimism amongst these groups that the forensic evidence collected through this application will be useful in the pursuit of justice and accountability for crimes of sexual violence. This study is unique in comparing the quality of data collected via a mobile application with data collected via paper-based forms and establishes important methodologies for evaluating these types of interventions going forward.

MediCapt data more frequently had higher data-quality scores than the paper-based forms, indicating that digital data collection of a standardized forensic medical certificate (in this case, the Kenyan PRC form) was of higher quality than data collected using a paper-based version of the same medical certificate. While end-user uptake is often presented as an obstacle to the implementation of mobile or digital technology over paper-based options, especially in low-resource environments, the results here suggest that uptake of these technologies was not, in fact, prohibitive in this context [30,31].

These results identify the use of a mobile application as a strategy that standardizes the collection of forensic evidence of sexual violence across different health facilities in the same jurisdiction. When comparing the quality of data collected using the paper-based forms, it was statistically higher in Nakuru compared to Naivasha. However, there was no statistical difference in quality in the MediCapt data across sites. An important feature of MediCapt that may support the increase in data quality is that incomplete fields are indicated to the user in the interface and on a summary page before completion of data collection. This feature serves as a reminder and an automatic data quality check built into the application. This is consistent with other studies, which have shown that routine data quality checks were associated with meaningful improvements in data quality of both paper and electronic medical records and increased concordance across multiple sites [32–34].

Although it is early to assess the impact of MediCapt on survivor outcomes, users were optimistic about its usefulness and reported that its legibility and photography features had already been appreciated by the court. The application’s required consent process also greatly increased the frequency of providers seeking consent, helped empower the survivors in their care, and improved the quality of documentation and survivor-centeredness of the sexual violence services. This finding is aligned with other literature that has shown that focusing on survivor empowerment and feminist-informed models of engaging with survivors for documentation of sexual violence can both improve survivor experiences and documentation outcomes [17,18]. The process for obtaining informed consent should be interpreted as a survivor-centered strength of MediCapt, as the application prompts clinicians to engage with survivors, empowers survivors to make decisions about the provision of their information to law enforcement and to decide about the collection of forensic photographs for their case, and requires clinicians to take great care in obtaining consent for every step in the evaluation, including the photographs.

It is important to note that informants did share some conflicting feedback related to the ease of use and acceptability of informed consent and consent for photography processes in MediCapt. Respondents spoke both about challenges with collecting consent on paper-based forms and concerns regarding obtaining survivor consent in MediCapt. Concerns for the consent process in MediCapt seem to more often relate to the time that it takes to obtain consent in a meaningful way through the application and the different steps in the examination and documentation process.

While, at least initially, using MediCapt may overall take slightly longer than using the paper form, the usability questionnaire and interviews suggested that this trend is reversed as providers became more familiar with the use of mobile technology and the MediCapt application specifically. This indicates that not only does MediCapt improve the quality of documentation collected but, over time, it improves the efficiency of forensic documentation, giving clinicians more time for interaction with patients to ensure survivor-centered care and appropriate referrals to different services and resources. Additionally, data from the usability questionnaire and semi-structured interviews suggest that use of MediCapt is acceptable to both clinicians and survivors, which can likely be linked to the improved efficiency in documentation. The use of mobile health applications has been shown to be time-saving and to increase the efficiency of patient information collection [35]. Technology can have a mixed impact on clinicians’ connection to or time spent with patients; while it increases efficiency, it should not be seen as synonymous with a reduction in time spent on patient care, particularly in cases of sexual violence [36]. This is why an emphasis is placed in MediCapt training on how clinicians can use technology with survivors in a way that is empowering and transparent.

Data security and privacy are issues that have been raised by end-users and stakeholders, especially because of the sensitive nature of the information and evidence collected. Among its key features, MediCapt includes sophisticated data encryption, high fidelity to chain of custody standards, and tamper-proof metadata. At the hardware level, end-users have unique credentials, passwords and PIN number that grant them access to the application and the forms they fill out. PHR works also with security experts to run audits to expose any potential gaps in the system ensuring that we proactively test and maintain the code. Currently the practice at most health facilities is that sensitive documentation, including paper PRC forms, are kept in the open, on desks, or stored in unlocked cabinets where any unauthorized individual can access them. MediCapt introduces additional levels of security and protection to ensure that these records are stored in a secured, encrypted space, allowing only those key individuals with passwords to access the sensitive information.

Major costs for MediCapt so far have included technology development for maintenance of the app and upgrading of the code and core implementation staff. Potential MediCapt costs for those implementing at the facility level will include technology maintenance expenses to facilitate the integration of MediCapt with existing systems for medical records, hospital costs for implementation materials (tables, printers, upgraded WiFi etc.) and capacity development expenses for training staff and administrators to engage with and effectively use MediCapt. There may be additional and ongoing resource needs including WiFi expenses and hardware such as tablets, printers, and ink. The results of this pilot are being used to understand how to deliver a cost-effective product at scale.

The results of this study show that MediCapt has the potential to dramatically improve how forensic evidence is collected in Kenya, and potentially in other low-resource settings at multiple levels: completeness, standardization, consent, and survivor-centeredness. Research has shown the need for strengthened tools and protocols to improve forensic documentation of sexual violence globally [37]. While few studies have documented the impact of forensic medical evidence in resource-constrained settings, one study found an association between documentation of anogenital injuries and convictions in South Africa [16]. Others have noted that the methods and practices for forensic evaluations more generally vary greatly amongst experts and centers [38]. MediCapt’s reliance on a digitized standardized forensic medical certificate helps to improve the quality of forensic evidence and to ensure that the data the clinicians document and share with police is focused on forensic medical evidence useful for police investigations and the legal process. Furthermore, the standardization of forensic evidence collection at the core of MediCapt can help support efforts to provide more survivor-centered care by ensuring evidence is complete and not requiring repeat and potentially retraumatizing interviews with survivors [39].

The data from this study are stored in the Physicians for Human Rights’ password-protected database which can only be accessed by the PHR Sexual Violence team. Due to the scope of participant consent, we request that the data not be available publicly, but can be accessed by PLOS One or other researchers, upon reasonable request, to validate our findings. To request for the data, please contact Dr. Michele Heisler at mheisler@phr.org, Medical Director and head of the ethics committee at Physicians for Human Rights.

MediCapt supports a broader range of trained health professionals in documenting high quality forensic evidence of sexual violence in contexts where those services are harder to access due to shortages in trained personnel [40]. MediCapt can contribute to survivors better realizing their right to health by ensuring that medical-legal examinations are more accessible, acceptable, available, and of higher quality [41]. Finally, survivors’ access to justice may also be improved in the sense that, with MediCapt, there is higher-quality, probative evidence that is gathered to support their cases. Against this background, MediCapt is poised to become a robust tool to address impunity for crimes of SGBV in low-resource settings. For MediCapt and other similar applications to achieve these intended outcomes, it is important that there is a continued collaborative design approach with multisectoral actors to achieve maximum functionality and responsiveness of the intervention for all stakeholders [24].

There were limitations to this study. Clinicians were asked about their experiences before and after MediCapt introduction and, as some time may have passed since the initial MediCapt training, this may have introduced recall or response bias. As a result of any reliance on self-reporting, there is also the possibility of social-desirability bias. However, this potential bias was minimized by using external evaluators, encouraging participants to share both the good and the bad, and informing participants that responses will remain anonymous and only reported in aggregate. This study was conducted at just two health care facilities–both located in one county in Kenya. While we do not see obvious differences between these facilities and others in Kenya or beyond, they may not be representative and, therefore, their findings cannot confidently be generalized to other settings. Future evaluations can be conducted in other areas where MediCapt is in use, such as in the Democratic Republic of the Congo. Future research could explore acceptability of MediCapt technology to survivors of sexual and gender-based violence, what factors eased uptake of the mobile application, and how added time with survivors was spent, given the increased efficiency gained from use of the application. Finally, it is critical to understand what effects improved forensic medical evidence of sexual violence have on prosecutions and case outcomes.

## Conclusions

MediCapt has been well received in Kenya across all sectors and has been shown to significantly improve the quality and standardization of forensic data collected across sites. It is anticipated that this improvement in forensic documentation will, most importantly, increase the likelihood of successful prosecutions, resulting in strengthening accountability for alleged perpetrators and improving access to redress and justice for survivors. An application like MediCapt has the ability to reduce impunity for crimes of sexual violence and ensure that a survivor-centered approach remains at the core of sexual violence response.

## Inclusivity in global research

Additional information regarding the ethical, cultural, and scientific considerations specific to inclusivity in global research is included in the Supporting Information (SX Checklist) in S4 File.

## Supporting information

S1 Fig MediCapt screenshots. (TIF)

S2 Fig MediCapt screenshots. (TIF)

S1 File Kenya Post-Rape Care form. (PDF)

S2 File MediCapt usability questionnaire. (PDF)

S3 File Semi-structured interview guide. (PDF)

S4 File Inclusivity in global research questionnaire. (DOCX)
