# Supplementary figures and images for: Evaluating the effectiveness of a mobile application to improve the quality, collection, and usability of forensic documentation of sexual violence
Source: PLoS One. 2022 Dec 14;17(12):e0278312. doi: 10.1371/journal.pone.0278312 (PMC9750009; doi:10.1371/journal.pone.0278312)

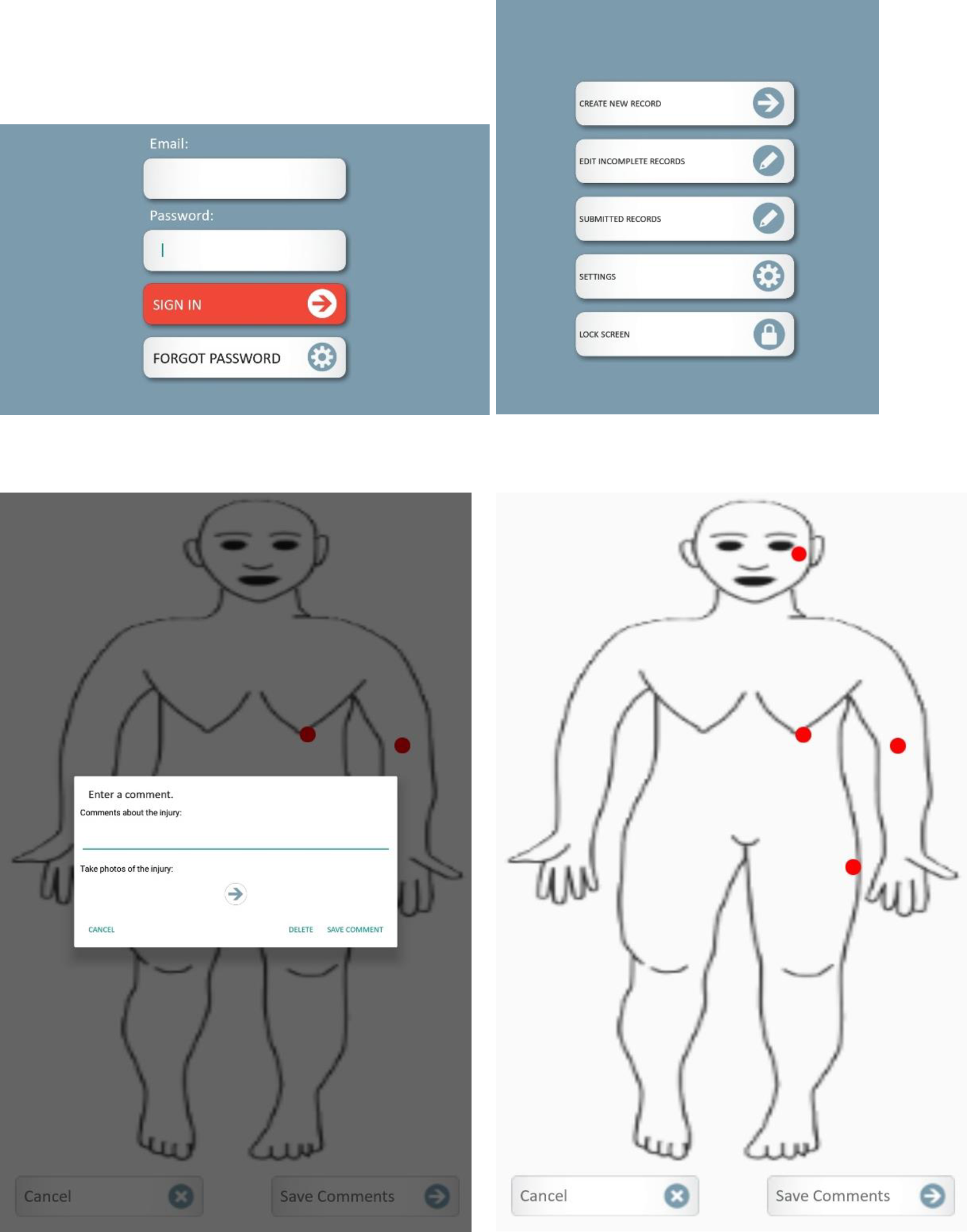

Supplement: S1 Fig — (TIF) [file pone.0278312.s001.tif]

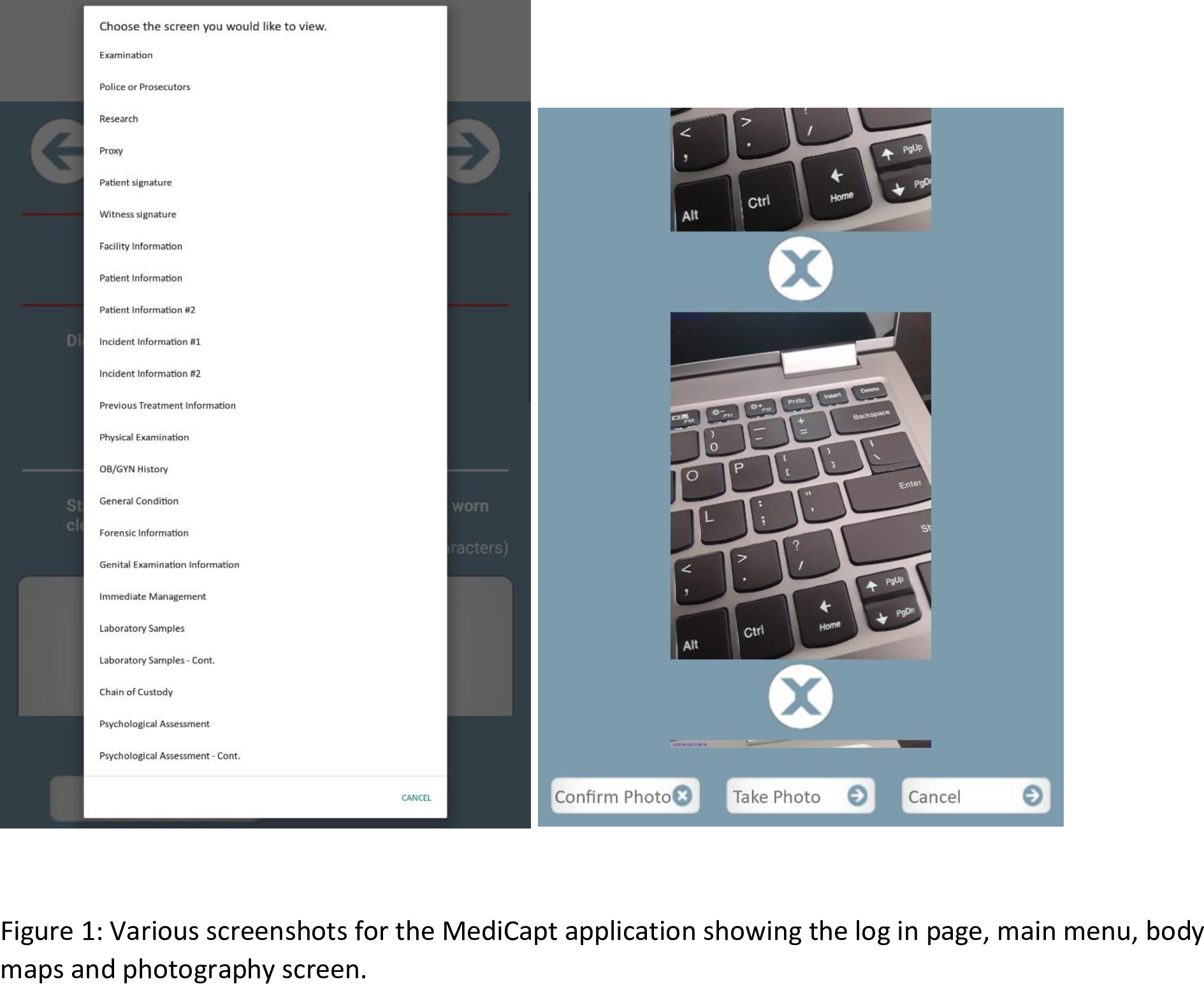

Supplement: S2 Fig — (TIF) [file pone.0278312.s002.tif]
