# Supplementary material for: Evaluating the effectiveness of a mobile application to improve the quality, collection, and usability of forensic documentation of sexual violence
Source: PLoS One. 2022 Dec 14;17(12):e0278312. doi: 10.1371/journal.pone.0278312 (PMC9750009; doi:10.1371/journal.pone.0278312)
Supplement: S1 File — (PDF) [file pone.0278312.s003.pdf]

MINISTRY OF HEALTH

POST RAPE CARE FORM (PRC)
MOH 363

PART A & B

County: ____

Sub-County: ____

Facility: ____

Start Date: ____________________ **End Date:** ____________________

POST RAPE CARE FORM (PRC)

PART A

PRC FORM IS **NOT** FOR SALE

PRC
Post Rape Care Form

MOH 363

Ministry of Health National Rape Management Guidelines: Examination documentation form for survivors of rape/sexual violence (to be used as clinical notes to guide filling in of the P3 form)

MOH 363

Date	Day	Month	Year	County Code	Sub-county Code / Facility Name	OP/IP No. / MFL Code

Name(s) (Three Names)	Date of birth	Day	Month	Year	☐ Male ☐ Female

Contacts (Residence and Phone number) ____________________

Disabilities (Specify) ____________________

Orphaned vulnerable child (OVC) ☐ Yes ☐ No

Marital Status (specify) ____________________

Citizenship ____________________

Date and time of Examination						Date and Time of Incident						No. of perpetrators
Day	Month	Year	Hr	Min	☐AM ☐PM	Day	Month	Year	Hr	Min	☐AM ☐PM	

Alleged perpetrators ☐ Male ☐ Female Estimated Age ____________

☐ Unknown ☐ Known (specify the relationship) ____________________

Where incident occurred

Administrative location: County ____________ Sub-county____________ Landmark____________

Chief complaints: Indicate what is observed ____________________

Indicate what is reported ____________________

Circumstances surrounding the incident (survivor account) remember to record penetration (how, where, what was used? Indication of struggle?)

____

____

Type of Sexual Violence

☐ Oral

☐ Vaginal

☐ Anal

☐ Other (specify) ____________

Use of condom? ☐ Yes ☐ No ☐ Unknown

Incident already reported to police? ☐ No ☐ Yes (indicate name of police station) ____________

Date and time of report	Day	Month	Year	Hr	Min	☐AM ☐PM

Attended a health facility before this one? ☐ No ☐ Yes (Indicate name of facility) ____________

Date	Day	Month	Year	Hr	Min	☐AM ☐PM

Were you treated? ☐ Yes ☐ No

Were you given referral notes? ☐ Yes ☐ No

Significant medical and/or surgical history

Comments: Indicate additional information provided by the client or observed by clinician

PHYSICAL EXAMINATION [indicates sites and nature of injuries bruises and marks outside the genitalia]
Please use the body map below to indicate injuries, inflammations, marks on various body parts of the survivor

BODY MAP

Anterior View

Posterior view

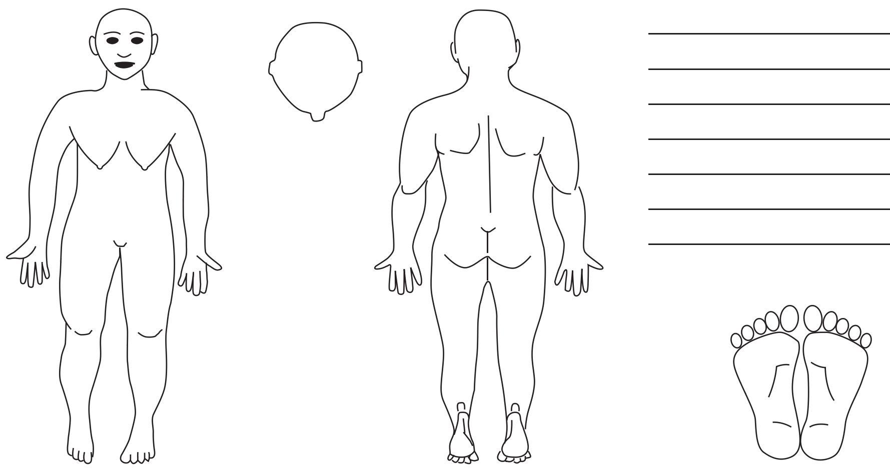

Comments

____

Female Genitalia

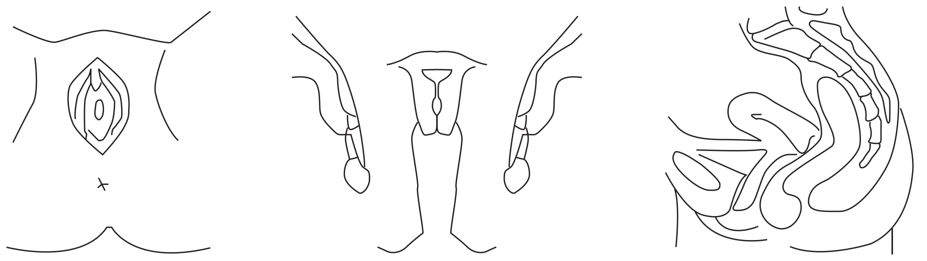

Male Genitalia

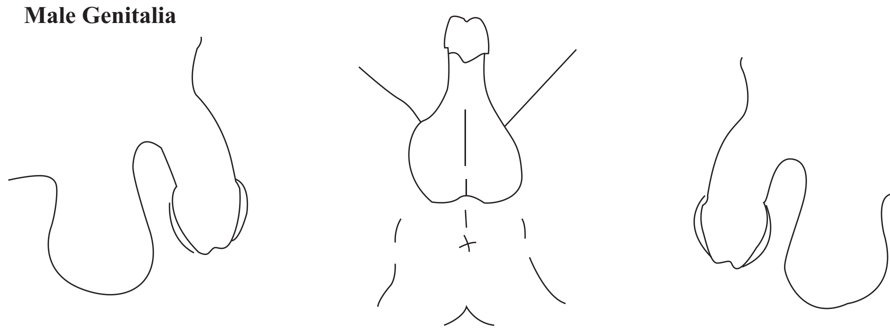

OB /GYN History	Parity	Contraception type	LMP	Known Pregnancy? ☐ Yes ☐ No	Date of last consensual sexual intercourse

General Condition	BP	Pulse Rate	RR	Temp	Demeanor /Level of anxiety (calm, not calm)

FORENSIC

Did the survivor change clothes? ☐ Yes ☐ No

State of clothes (stains, torn, color, where were the worn clothes taken)?

How were the clothes transported? ☐ a) Plastic Bag ☐ b) Non Plastic Bag ☐ c) Other (Give details) ____________________

Were the clothes handed to the police? ☐ Yes ☐ No

Did the survivor go to the toilet? ☐ Long call? ☐ Short call?

Did the survivor have a bath or clean themselves? ☐ No ☐ Yes (Give details)____________________

Did the survivor leave any marks on the perpetrator? ☐ No ☐ Yes (Give details) ____________________

GENITAL EXAMINATION OF THE SURVIVOR-indicate discharges, inflammation, bleeding

Describe in detail the physical status

Physical injuries (mark in the body map) ____________________

Outer genitalia ____________________

Vagina ____________________

Hymen ____________________

Anus ____________________

Other significant orifices ____________________

Comments

____

Immediate Management	PEP 1st dose	ECP given	Stitching /surgical toilet done	STI treatment given
	☐ No	☐ No	☐ No	☐ No
	☐ Yes (No of tablets)	☐ Yes	☐ Yes(Comment) ____________	☐ Yes(Comment) ____________

Any other treatment / Medication given /management?

Referrals to

☐ Police Station ☐ HIV Test ☐ Laboratory ☐ Legal ☐ Trauma Counseling

☐ Safe Shelter ☐ OPD/CCC/HIV Clinic ☐ Other (specify)

LABORATORY SAMPLES	Sample Type	Test	Please tick as is applicable: National government Lab	Health Facility Lab	Comments
	Outer Genital swab<br>Anal swab<br>Skin swab<br>Oral swab<br>Specify	Wet Prep Microscopy			
		DNA			
		Culture and sensitivity			
	High vaginal swab	Wet Prep Microscopy			
	Urine	Pregnancy Test			
		Microscopy			
		Drugs and alcohol			
		Other			
	Blood	Haemoglobin			
		HIV Test			
		SGPT/GOT			
		VDRL			
		DNA			
	Pubic Hair	DNA			
	Nail clippings	DNA			
	Foreign bodies	DNA			
	Other (specify)				

CHAIN OF CUSTODY

These /All / Some of the samples packed and issued (please specify)

			Day	Month	Year
By	Name of Examining Officer (Doctor/Nurse/Clinical officer)	Signature			
To	Police Officer's Name	Signature			

PSYCHOLOGICAL ASSESSMENT Complete psychological assessment section in Part B

POST RAPE CARE FORM (PRC)

PRC FORM IS **NOT** FOR SALE

PART B

PSYCHOLOGICAL ASSESSMENT

PRC
Post Rape Care Form

MOH 363

Part B is intended to assess the mental status of a client in order to be able to offer holistic care. This should inform the management and subsequent follow up of the client and hence should be filled in at presentation.

Psychological assessment should be done by trained health care providers including Medical Officers, Nurses, Clinical Officers, Psychiatrists, Psychological Counselors and Medical Social Workers duly recognized by the Ministry of Health.

The Medical Officers and other persons designated by law as expert witnesses in court (Nurses and Clinical Officers) should be the ones to sign off both the Part A and B of the PRC form.

General appearance and behavior
Note appearance (appear older or younger than stated age), gait, dressing, grooming (neat or unkempt) and posture.

____

____

____

Rapport
Easy to establish, initially difficult but easier over time, difficult to establish.

____

____

____

Mood
How he/she feels most days (happy, sad, hopeless, euphoric, elevated, depressed, irritable, anxious, angry, easily upset).

____

____

____

Affect
Physical manifestation of the mood e.g. labile (emotions that are freely expressed and tend to alter quickly and spontaneously like sobbing and laughing at the same time), blunt/ flat, appropriate/ inappropriate to content.

____

____

____

Speech
Rate, volume, speed, pressured (tends to speak rapidly and frenziedly), quality (clear or mumbling), impoverished (monosyllables, hesitant).

____

____

____

Perception
Disturbances e.g. Hallucination, feeling of unreality (corroborative history may be needed to ascertain details)

____

____

____

Thought content
Suicidal and Homicidal Ideation (Ideas but no plan or intent; clear/unclear plan but no intent; ideas coupled with clear plan and intent to carry it out); any preoccupying thoughts.

____

____

____

Thought process
Goal-directed/ logical ideas, loosened associations/ flight of ideas/ illogical, relevant, circumstantial (drifting but often coming back to the point), ability to abstract, perseveration (constant repetition, lacking ability to switch ideas).

____

____

____

(For children use wishes and dreams, and art/ play therapy to assess the thought process and content.
-Through drawing and play (e.g. use of toys). Allow the child to comment on the drawing and report verbatim.

____

____

____

-Assess the unconscious world of the child by asking about feelings e.g. ask the child to report the feeling that he/she commonly experiences and ask what makes him/her feel that way

____

____

____

Cognitive function-

a. Memory: Recent memory, long-term and short term memory (past several days, months, years).

____

____

____

b. Orientation: to time, place, person i.e. ability to recognize time, where they are, people around e.t.c.

____

____

____

c. Concentration: ability to pay attention e.g. counting or spelling backwards, small tasks

____

____

____

d. Intelligence: Use of vocabulary (compare level of education with case presentation; above average, average, below average).

____

____

____

e. Judgment: Ability to understand relations between facts and to draw conclusions; responses in social situations.

____

____

____

Insight level: Realizing that there are physical or mental problems; denial of illness, ascribing blame to outside factors; recognizing need for treatment (Indicate whether insight level is; present, fair, not present)

____

____

____

Recommendation following assessment	Referral point/s

Referral uptake since last visit e.g. other medical services, children's department, police, legal aid, shelter e.t.c.

____

____

			Day	Month	Year
By	Name of Examining Officer (Doctor/Nurse/Clinical officer)	Signature			
To	Police Officer's Name	Signature	Day	Month	Year
