# Supplementary material for: Evaluating the effectiveness of a mobile application to improve the quality, collection, and usability of forensic documentation of sexual violence
Source: PLoS One. 2022 Dec 14;17(12):e0278312. doi: 10.1371/journal.pone.0278312 (PMC9750009; doi:10.1371/journal.pone.0278312)
Supplement: S2 File — (PDF) [file pone.0278312.s004.pdf]

MediCapt Usability & Feasibility Questionnaire

Version: April 2021

The following questionnaire contains items that help us evaluate the technology we are developing and the pilot overall. This questionnaire is anonymous and your answers will not be shared with anyone outside of PHR and its evaluation team. Your responses will have no effect on your employment or relationship with your place of work, PHR personnel and consultants.

A. Which of the following best describes your occupation?

___Nurse
___Doctor
___Other, please specify: ____

B. If you have a clinical specialty, please describe it in the field below.

C. How many years have you been conducting sexual assault examinations?

I have been conducting sexual assault exams for __________ years.

D. How many sexual assault cases have you entered into MediCapt?

I have entered ___________ cases into MediCapt.

E. Approximately how many sexual assault examinations do you typically conduct in one month? If you do not know the exact number, please provide your best estimate.

I conduct approximately _________ sexual assault examinations per month.

F. Approximately how much time do you spend per sexual assault EXAMINING THE SURVIVOR (not including documentation)? If you do not know the exact time, please provide your best estimate.

I spend approximately _________ minutes per sexual assault EXAMINING.

G. Approximately how much time do you spend per sexual assault DOCUMENTING USING THE PAPER FORM? If you do not know the exact time, please provide your best estimate.

I spend approximately _________ minutes per sexual assault DOCUMENTING USING THE PAPER FORM.

H. Approximately how much time do you spend per sexual assault DOCUMENTATION using MediCapt? If you do not know the exact time, please provide your best estimate.

I spend approximately _________ minutes per sexual assault DOCUMENTING USING MEDICAPT.

I. Have you ever used a mobile phone?

___Yes
___No

J. Have you ever used a smart phone? (A "smart phone" is a mobile phone that performs many of the functions of a computer, typically having a touchscreen interface, internet access, and an operating system)

___Yes
___No → IF NO, PLEASE SKIP TO QUESTION N

K. If you have experience using a smart phone, what have you used it for? Check all that apply.

___Communicating with family and friends
___Looking up information online
___To check email
___To take pictures
___To assist with my clinical work
___To find information to make medical decisions
___To take notes
___To play games
___To use apps
___To listen to music

L. Do you have experience using applications, or "apps", on smart phones? (e.g., camera, WhatsApp, Viber, ViuSasa, Truecaller)

___Yes
___No → IF NO, PLEASE SKIP TO QUESTION N
If yes, which apps have you used?

M. Have you ever used the camera function on a mobile phone or smart phone?
___Yes
___No

N. Have you ever used a digital camera (not on a mobile or smart phone) to take a photograph?
___Yes
___No

O. Do you normally take forensic photographs when you conduct sexual assault examinations (before using MediCapt)?
___Yes
___No

P. Below, please indicate the extent to which you agree with each of the statements.

	I strongly disagree	I disagree	I agree	I strongly agree	Comment (optional)
TABLETS – The following questions regard using MediCapt on a **tablet device**. Please answer the questions as they pertain to using the tablet.					
1. The tablet itself appears to be suitable to document sexual assault examinations.	☐	☐	☐	☐	
2. It is easy to type on the tablet.	☐	☐	☐	☐	
3. It is easy to use the touchscreen on the tablet.	☐	☐	☐	☐	
4. It is easy for me to hold the tablet.	☐	☐	☐	☐	
5. The tablet should be smaller.	☐	☐	☐	☐	
6. It is easy to take photographs using the tablet.	☐	☐	☐	☐	
7. It is easy to connect the Bluetooth keyboard to the tablet.	☐	☐	☐	☐	
8. It is easy to type using the keyboard.	☐	☐	☐	☐	
**USABILITY OF MEDICAPT. The following questions concern the MediCapt app itself.**					
9. The screens appear to be straightforward and easy to use.	☐	☐	☐	☐	
10. I find it easy to transition from one screen to the next.	☐	☐	☐	☐	
11. I find the text size on the screens too small.	☐	☐	☐	☐	

12. I like the colors used on the screens for MediCapt.	☐	☐	☐	☐	
13. I find that multiple places to enter information on a single screen makes data entry on MediCapt easy to use.	☐	☐	☐	☐	
14. I find the pictogram easy to use.	☐	☐	☐	☐	
15. The different screens all made sense to me.	☐	☐	☐	☐	
16. It was easy for me to use MediCapt.	☐	☐	☐	☐	
17. I like the prompts to take forensic photographs that were built into MediCapt.	☐	☐	☐	☐	
18. MediCapt offers a useful way to take forensic photography.	☐	☐	☐	☐	
**APPROPRIATENESS OF MEDICAPT**					
19. MediCapt helps me do a better job of documenting sexual assault examinations.	☐	☐	☐	☐	
20. MediCapt helps me save time in conducting sexual assault examinations.	☐	☐	☐	☐	
21. It is easy to use MediCapt while I am conducting a sexual assault examination on a patient.	☐	☐	☐	☐	
22. It is easier for me to take forensic photographs using MediCapt.	☐	☐	☐	☐	
23. My patients will be better served if I use MediCapt.	☐	☐	☐	☐	
24. Using MediCapt makes a difference in survivor's cases.	☐	☐	☐	☐	
25. Printing the MediCapt document serves the patient well.	☐	☐	☐	☐	
**ACCEPTABILITY OF MEDICAPT**					

26. I currently complete a paper-based medical certificate for examinations of all sexual violence patients.	☐	☐	☐	☐	
27. The risks to the patient of lost personal information are greater with the paper form than with MediCapt.	☐	☐	☐	☐	
28. I am comfortable using MediCapt in my clinical practice.	☐	☐	☐	☐	
29. I think that sexual violence patients accept my use of MediCapt during their examination.	☐	☐	☐	☐	
30. The use of MediCapt with a sexual violence patient is culturally unacceptable.	☐	☐	☐	☐	
31. The training on using MediCapt with the patient helped me incorporate it into practice.	☐	☐	☐	☐	
32. I like to use new types of technology to help my patients.	☐	☐	☐	☐	
33. I think that the forensic photography function on MediCapt makes it more comfortable for my patients to be photographed, versus using a separate camera to take photographs.	☐	☐	☐	☐	
34. I believe that my patients understand the risks and benefits of using MediCapt.	☐	☐	☐	☐	
35. I am obtaining the consent of all patients prior to using MediCapt.	☐	☐	☐	☐	
36. Patients readily provide consent for MediCapt to be used in documenting their cases.	☐	☐	☐	☐	
37. The process of obtaining consent to use MediCapt for data collection is too cumbersome.	☐	☐	☐	☐	
38. I feel confident in my ability to explain to the patient the purpose and risks involving the use of MediCapt to obtain and record their information.	☐	☐	☐	☐	
39. Printing the MediCapt document is more acceptable to me than sending the data electronically.	☐	☐	☐	☐	

40. Overall, I am satisfied with MediCapt.	☐	☐	☐	☐	
**FEASIBILITY AND SUSTAINABILITY OF MEDICAPT**					
41. MediCapt is intuitive to my needs when documenting sexual assault examinations.	☐	☐	☐	☐	
42. The MediCapt app "made sense."	☐	☐	☐	☐	
43. My colleagues will be happy using MediCapt.	☐	☐	☐	☐	
44. I have had enough training to use MediCapt correctly.	☐	☐	☐	☐	
45. The device is likely to get stolen.	☐	☐	☐	☐	
46. I am likely to lose my device.	☐	☐	☐	☐	
47. Additional measures will need to be put into place to make sure this device gets used.	☐	☐	☐	☐	
48. I could one day train my colleagues on how to use MediCapt to document sexual assault examinations.	☐	☐	☐	☐	
49. MediCapt helps me save time in documentation.	☐	☐	☐	☐	
50. MediCapt will ensure that sexual assault records are transferred to the appropriate law enforcement and legal personnel.	☐	☐	☐	☐	
51. MediCapt is better than what I am currently using to document sexual assaults.	☐	☐	☐	☐	
52. I am able to take forensic photographs easily using MediCapt.	☐	☐	☐	☐	
53. Healthcare professionals who use MediCapt will take better forensic photographs because they are using MediCapt.	☐	☐	☐	☐	

54. It is difficult to get reliable Wi-Fi or internet access to transmit the files.	☐	☐	☐	☐	
55. I have Wi-Fi or other internet access in my community or health care center.	☐	☐	☐	☐	
56. I think connection to the internet is a major problem for uploading files.	☐	☐	☐	☐	
57. I have access to reliable electricity in my community or health care center.	☐	☐	☐	☐	
58. It is to charge the smart phones or tablets on a daily basis.	☐	☐	☐	☐	
59. I have to rely on a generator to charge smart phones or tablets on a daily basis.	☐	☐	☐	☐	
60. It is easy to troubleshoot problems that I encounter with MediCapt.	☐	☐	☐	☐	
61. When I encounter a problem with MediCapt I know who to turn to for help.	☐	☐	☐	☐	
62. When I encounter a problem with MediCapt, I am satisfied with the help I receive.	☐	☐	☐	☐	
63. The printing process with MediCapt works well.	☐	☐	☐	☐	
64. It is difficult to maintain printer supplies (ink, paper, etc.).	☐	☐	☐	☐	
65. The printer is likely to be stolen.	☐	☐	☐	☐	
66. The printer is likely to be used by others for purposes UNRELATED to MediCapt.	☐	☐	☐	☐	

Q. Please describe your experience in using MediCapt (in 4 sentences or less).

R1. What problems, if any, did you have using MediCapt?

R2. If you encountered problems when using MediCapt, what did you do? If you reached out to a support person, please describe that experience.

S. What were your favorite aspects of the MediCapt app?

T. What additional measures will be needed for you to use MediCapt?

U1. If you are already using MediCapt with patients, please describe how you obtain patient consent.

U2. Please describe patients' general response in providing consent. Please describe any challenges you may be experiencing in obtaining patients' consent.

V. What else could we do in order to improve the MediCapt app for your needs?

W. Can you think of one particular case where MediCapt has made a difference in the case of a survivor of sexual assault? If so, please share below.

Thank you for taking the time to complete this assessment. Please turn the assessment to a PHR staff person once you have completed it.
