# Supplementary material for: Evaluating the effectiveness of a mobile application to improve the quality, collection, and usability of forensic documentation of sexual violence
Source: PLoS One. 2022 Dec 14;17(12):e0278312. doi: 10.1371/journal.pone.0278312 (PMC9750009; doi:10.1371/journal.pone.0278312)
Supplement: S3 File — (PDF) [file pone.0278312.s005.pdf]

PROGRAM ON SEXUAL VIOLENCE IN CONFLICT ZONES

[The baseline assessment focused on the interviewees' experience using the paper records. The final assessment will focus on interviewees' experience using MediCapt.]

Semi-structured interview guide for clinicians and non-clinicians related to MediCapt

Version: October 2020

Date	|___||___| / |___||___| / |___||___| *(dd/mm/yyyy)*
Primary Interviewer Name	
Country	
Location of interview	
Clinician or non-clinician user	
Audio record number	
Translator	

INTERVIEWER: EXPLAIN TO THE PARTICIPANT THAT YOU ARE INTERESTED IN HEARING THEIR CANDID THOUGHTS ON THE MEDICAPT APP FOR DOCUMENTING MEDICAL EVIDENCE OF SEXUAL VIOLENCE AND ASSURE THEM THAT ALL OF THEIR RESPONSES WILL BE KEPT ANONYMOUS AND ONLY REPORTED IN AGGREGATE WITH OTHER PARTICIPANTS. PLEASE READ VERBAL CONSENT SCRIPT, AND SIGN/DATE CONSENT STATEMENT BEFORE PROCEEDING, AND THEN PLEASE WRITE YOUR INITIALS HERE: ________

A. BACKGROUND

1. What is your professional position?

2. How long have you been working with this organization/agency/unit?

3. [For clinical and non-clinical interviewees at healthcare facilities…] At your institution, how many clinicians work with survivors of sexual violence?

   How many of those clinicians currently use the MediCapt application?

4. Please describe any training, refresher training, or formal briefing you have received regarding use of the MediCapt app. When was that training?

Now I am going to ask you some questions about your experiences using the MediCapt application.

INTERVIEWERS – GO TO SECTION:

B CLINICIAN USERS

C NON-CLINICIAN USERS AT HEALTH FACILITIES (e.g., admin, IT, records)

D LAW ENFORCEMENT PERSONNEL

B. QUESTIONS FOR CLINICIAN USERS

Paper-based records (questions for all participants):

1. What are your thoughts on the use of paper-based PRC form documentation for sexual violence cases?

2. What works well with paper-based PRC form documentation?

3. What doesn't work well with paper-based PRC form documentation?

4. Can you provide an example or two when any shortcomings in paper-based documentation negatively impacted a survivor's case? How common is this?

5. How common is it for paper-based documentation to go missing sometime between when a survivor initially presents and their case is decided in the courts?

6. Would [or does] digital documentation address some of these problems with paper-based documentation? In which ways?

7. Is there anything else you'd like to share or discuss with us?

MediCapt (if participant has MediCapt experience):

1. Please describe how you are currently using the MediCapt application.

2. What are the primary problems you have experienced in using the MediCapt application?

3. What do you do when you encounter a problem?

4. When you have a problem or something goes wrong with MediCapt, who do you call? Who do you email?

5. When something goes wrong with MediCapt and you seek assistance with the problem, how would you describe the responsiveness to your problem?

6. Are you currently obtaining patient consent before using the MediCapt application? Always, sometimes, never?

7. Can you describe the patients' reaction to the process of obtaining consent? Has anyone declined consent for any part of the process? What has been your experience obtaining consent for forensic photography? Have patients always consented to the forensic photography with the MediCapt app?

8. Have you been having any problems (technical difficulties or issues with explaining the material to your patients) with the process of obtaining consent?

9. What are the benefits of using MediCapt?

10. What differences do you see with MediCapt in comparison to the paper form?

11. In your view, what are some of the ways that the MediCapt application could be improved?

12. Can you provide one example of a survivor of sexual assault whose experiences or outcomes were different as a result of the MediCapt application?

13. Do you feel that using the MediCapt application has produced any unintended consequences? Please describe any positive or negative unintended consequences.

14. Is there anything else you'd like to share or discuss with us?

C. QUESTIONS FOR NON-CLINICIAN USERS (e.g., admin, IT, medical records)

Paper-based records (questions for all participants):

1. What are your thoughts on the use of paper-based PRC form documentation for sexual violence cases?

2. What works well with paper-based PRC form documentation?

3. What doesn't work well with paper-based PRC form documentation?

4. Can you provide an example or two when any shortcomings in paper-based documentation negatively impacted a survivor's case? How common is this?

5. How common is it for paper-based documentation to go missing sometime between when a survivor initially presents and their case is decided in the courts?

6. Would [or does] digital documentation address some of these problems with paper-based documentation? In which ways?

MediCapt (if participant has MediCapt experience):

1. Please describe how you are currently using or supporting the MediCapt application.

2. What are the primary problems you have experienced in using the MediCapt application?

3. When you have a problem or something goes wrong with MediCapt, who do you call? Who do you email?

4. When something goes wrong with MediCapt and you seek assistance with the problem, how would you describe the responsiveness to your problem?

5. What are the benefits of using MediCapt?

6. What differences do you see with MediCapt in comparison to the paper form?

7. In your view, what are some of the ways that the MediCapt application could be improved?

8. Do you feel that using the MediCapt application has produced any unintended consequences? Please describe any positive or negative unintended consequences.

9. Is there anything else you'd like to share or discuss with us?

Those are all the questions we have. Thank you for your time.

D. QUESTIONS FOR LAW ENFORCEMENT PERSONNEL

Paper-based records (questions for all participants):

1. What are your thoughts on the use at health facilities of paper-based PRC form documentation for sexual violence cases?

2. What works well with paper-based PRC form documentation?

3. What doesn't work well with paper-based PRC form documentation?

4. Can you provide an example or two when any shortcomings in paper-based documentation negatively impacted a survivor's case? How common is this?

5. How common is it for paper-based documentation to go missing sometime between when a survivor initially presents and their case is decided in the courts?

6. Would [or does] digital documentation address some of these problems with paper-based documentation? In which ways?

MediCapt (if participant has MediCapt experience):

1. Please describe your understanding and experience with the MediCapt application.

2. What are the primary problems you have seen with the MediCapt application?

3. What are the benefits of using MediCapt?

4. How do you think MediCapt could impact your work in law enforcement? Do you think MediCapt could improve legal outcomes for survivors of sexual violence?

5. Can you provide one example of a survivor of sexual violence whose experiences or outcomes were different as a result of the MediCapt application?

6. What differences do you see with MediCapt in comparison to the paper form?

7. In your view, what are some of the ways that the MediCapt application could be improved?

8. Have you received forensic photographs documented through MediCapt? If so, what impact (if any) did the photographs have on your work and/or the cases?

9. Do you feel that using the MediCapt application has produced any unintended consequences? Please describe any positive or negative unintended consequences.

10. Is there anything else you'd like to share or discuss with us?

Those are all the questions we have. Thank you for your time.
